# Cow Milk and Intestinal Epithelial Cell-Derived Extracellular Vesicles as Systems for Enhancing Oral Drug Delivery

**DOI:** 10.3390/pharmaceutics12030226

**Published:** 2020-03-04

**Authors:** Greta Carobolante, Julia Mantaj, Enrico Ferrari, Driton Vllasaliu

**Affiliations:** 1 School of Cancer and Pharmaceutical Sciences, Faculty of Life Sciences & Medicine, King’s College London, London SE1 9NH, UK; greta.carobolante@studenti.unipd.it (G.C.); julia.mantaj@kcl.ac.uk (J.M.); 2 Department of Pharmaceutical and Pharmacological Sciences, Università degli Studi di PADOVA, Via 8 Febbraio, 2, 35122 Padova, Italy; 3 School of Life Sciences, University of Lincoln, Lincoln LN6 7TS, UK; EFerrari@lincoln.ac.uk

**Keywords:** drug absorption, exosomes, extracellular vesicles, intestinal permeability, oral drug bioavailability

## Abstract

Ingestion is the preferred way for drug administration. However, many drugs have poor oral bioavailability, warranting the use of injections. Extracellular vesicles (EVs) from cow milk have shown potential utility in improving oral drug bioavailability. However, EVs produced by intestinal epithelial cells have not been investigated for this application. We compared the capacity of cow milk EVs and intestinal epithelial cell-derived counterparts to enhance oral drug bioavailability. EVs were isolated, fluorescently labelled, and loaded with curcumin (CUR) as a model poorly absorbable drug. These were then characterised before testing in an intestinal model (Caco-2). Epithelial cell-derived EVs showed notably higher cell uptake compared to cow milk EVs. Cell uptake was significantly higher in differentiated compared to undifferentiated cells for both types of EVs. While both milk- and cell-derived EVs improved the cell uptake and intestinal permeability of CUR (confirming oral drug bioavailability enhancement potential), epithelial cell EVs demonstrated a superior effect.

## 1. Introduction

The oral route is the preferred method of drug administration [1,2,3,4]. However, many drugs, including poorly water-soluble small drug molecules and macromolecular biologics (peptides, therapeutic proteins and nucleic acid-based therapies), have low oral bioavailability. This often necessitates the use of injection as the only clinically effective administration route. However, in addition to pain and potential injection site injury, injections frequently require administration by trained personnel, which increases treatment costs.

Because of the many benefits offered by oral drug administration, pharmaceutical strategies that safely and effectively improve oral drug bioavailability are highly desirable. As part of these strategies, vesicle- or particle-based nanoscale drug carriers have been studied for some time. This includes polymeric nanoparticles [5], liposomes [6,7] and solid-lipid nanoparticles [6,8]. However, these systems tend to suffer from inefficient translocation across the highly effective and selective barrier of the intestinal mucosa, therefore requiring careful formulation. Drug nanocarrier systems utilised to improve oral drug bioavailability can be designed to target epithelial biological transport pathways [7,9,10,11]. While some of these systems have shown potential in vitro and in animal studies, current understanding of orally-administered nanomedicine behaviours and biotransformation within the complex environment of the gastrointestinal tract (including interaction with gut biofluid and mucus, as well as potential toxicity) is lacking. Furthermore, it is currently unclear whether the transport capacity of such systems in vivo is adequate to ensure clinically relevant improvements in drug bioavailability. 

Extracellular vesicles (EVs) are different types of small-sized membrane vesicles or particles (including exosomes, microvesicles, apoptotic bodies, and virus-like particles) secreted by cells. EVs play a crucial role in intercellular communication by transferring information in the form of proteins, nucleic acids, and lipids from one cell to another present in close proximity or distant sites. EVs are secreted by all cell types and are present in biofluids, including blood, saliva, urine, cerebrospinal fluid and breast milk, and others [12]. As part of their key role of cargo transfer between cells, EVs clearly demonstrate an ability to cross biological barriers. Because of this property, EVs potentially offer significant advantages as drug delivery systems and have been extensively researched for this application in the past decade. 

Exosomes—a type of EV—have in particular attracted significant attention in drug delivery. Exosomes harvested from cow milk, which are interesting because of scalability potential [13], have been shown to be transported by human endothelial cells and exhibit cross-species tolerance without eliciting immune responses [14,15]. Importantly, cow milk exosomes have shown potential for improving oral drug bioavailability [15,16,17]. This potential rests on the demonstrated ability of cow milk exosomes to traverse the human intestinal epithelial barrier. However, the intestinal-permeating efficiency of these systems is currently unclear. The present study for the first time compared the intestinal translocation (and therefore oral drug delivery potential) of cow milk EVs with that of epithelial cell derived EVs. EV were initially isolated and characterised, prior to fluorescent labelling (to enable quantitation) and encapsulation, of curcumin (CUR) as a model of a poorly absorbed drug. Cell uptake of bare and CUR-loaded EVs was tested in undifferentiated and differentiated intestinal Caco-2 cells (gold standard in vitro intestinal model). Finally, EV permeability across Caco-2 monolayers was determined with or without CUR encapsulation. The study sheds further light into the potential utility of EVs, including those from edible sources, as natural systems for improving oral drug delivery. 

## 2. Materials and Methods 

Dulbecco’s Modified Eagle’s Medium (DMEM) with 4.5 g/L glucose, L-glutamine, sodium pyruvate and sodium bicarbonate, Hank’s Balanced Salt Solution (HBSS) modified with sodium bicarbonate (without phenol red), non-essential amino acids (NEAA), antibiotic/antimycotic solution (penicillin, streptomycin, and amphotericin) and foetal bovine serum (FBS, non-USA origin), Curcumin (CUR), Triton X-100 and QuantiPro^TM^ BCA Assay Kit were purchased from Sigma-Aldrich (Poole, UK). Caco-2 cells were purchased from the European Collection of Cell Cultures (ECACC) and used between passages p66–p74. MTS reagent, namely 3-(4,5-dimethylthiazol-2-yl)-5(3-carboxymethonyphenol)-2-(4-sulfophenyl)-2H-tetrazolium (commercially known as CellTiter 96 AQueous One Solution Assay) was purchased from Promega (Madison, WI, USA). Exosome isolation kit from other body fluids and exosome isolation kit form cell culture were purchased from Thermo Fisher Scientific (Loughborough, UK), ExoGlow™-Protein EV Labeling Kit (Red) and exosome-depleted FBS were purchased from System Biosciences (Palo Alto, CA, USA). Transwell^®^ permeable cell culture inserts (polycarbonate filter, 1.1 cm^2^ diameter, 0.4 μm pores) were obtained from Corning (Corning, NY, USA). PD-10 columns, Sephadex G-25 were obtained from GE Healthcare (Chicago, IL, USA). 

### 2.1. Isolation of EVs

EVs were isolated from cow skimmed milk obtained from a local grocery store, and from Caco-2 cells. We used a commercial kit for EV isolation, similarly to recent studies [18,19]. Milk derived EVs were isolated from 1 mL of milk according to the instructions supplied with the isolation reagent namely Invitrogen™ Total Exosome Isolation Reagent (from other body fluids). For Caco-2 EVs, cells were firstly grown in T75 flasks using DMEM supplemented with exosome-depleted FBS, antibiotic/antimycotic and NEAA. The flask was maintained under atmosphere set at 5% CO_2_, 95% relative humidity and 37 °C in an incubator. Cells were cultured until they were approximately 90% confluent, exchanging culture medium every two days. Culture medium was collected on confluence and EVs isolated according to the protocol enclosed within Invitrogen™ Total Exosome Isolation Reagent (from cell culture media). 

### 2.2. Characterisation of EVs

Characterization of both milk- and Caco-2 cell-derived EVs was performed by measuring the average hydrodynamic size by dynamic light scattering (DLS), zeta potential and polydispersity by a Malvern Zetasizer (Malvern instruments Ltd., Malvern, UK). The yield (EV particle number) was measured by nanoparticle tracking analysis (NTA) using a Nanosight LM10 instrument (Malvern, UK). Briefly, for DLS analysis a 1:100 dilution in phosphate buffered saline (PBS) of EVs was analysed in equilibration time of 120 s at a constant temperature of 25 °C. For NTA analysis, EVs diluted 1:100 dilution in PBS were analysed at a constant temperature of 25 °C. In both analyses, each measurement was repeated three times.

Bicinchoninic acid (BCA) protein assay was used to calculate EV proteins present in the solution after the isolation. 10 µL of EV suspension (diluted 1:100 in PBS) was used for protein quantitation and absorbance at 562 nm was measured and compared with the serially diluted bovine serum albumin standards. A calibration curve was plotted using standard solutions of BCA in the range 0–30 µg/mL and the concentration of the sample was measured. 

### 2.3. Fluorescence Labelling of EVs

Isolated EVs were labelled using a commercially available ExoGlow™-protein EV labelling kit. Briefly, 1 µL of 500X labelling dye per 200–500 µg of protein equivalent of EVs was added. The solution was incubated for 20 min at 37 °C with shaking. Then, 167 µL of ExoQuick™ reagent was added to the solution and incubated overnight at 4 °C. After incubation, the solution was centrifuged at 12,300× *g* for 10 min and EVs in the pellet resuspended in PBS for downstream applications. 

### 2.4. Curcumin Incorporation into EVs 

CUR was encapsulated into EVs by mixing a solution of CUR in ethanol (2 mg/mL) with EVs in PBS overnight at room temperature under stirring. The theoretical concentration ratio of CUR to EV proteins (determined by BCA assay) was kept 1:4. After the incubation, a size exclusion chromatography with PD-10 Sephadex G-25 column (Chicago, IL, USA). was conducted in order to remove the unincorporated curcumin from EV suspension. Elution profiles of CUR alone and EVs alone were used as controls. 

The loading capacity and the entrapment efficiency were then calculated using Equations (1) and (2), respectively:

(1) $$\%\;Drug\;loading=\frac{amount\;of\;CUR\;in\;EV-CUR}{amount\;of\;EV\;proteins\;in\;EV-CUR}\times 100$$

(2) $$\%\;Entrapment\;efficiency=\frac{amount\;of\;CUR\;in\;EV-CUR}{CUR\;added}\times 100$$

### 2.5. Cell Uptake f Eovs in Undifferentiated and Differentiated Caco-2 Cells 

Cell uptake of EVs was tested in undifferentiated Caco-2 cells (cultured in multiwell plates) and differentiated, polarised monolayers (cultured on inserts) to determine whether Caco-2 cell differentiation influences the extent of EV internalisation. 

For the uptake in undifferentiated cells, Caco-2 cells were seeded at 5 × 10^4^ cells/well in 48-well plates and cultured for 72 h. This was followed by culture medium removal and replacement with 0.5 mL of HBSS to equilibrate the cells (45 min incubation). Cells were incubated with EVs applied in HBSS for 4 h. After incubation, cells were detached from wells with trypsin and permeabilised using Triton-X 1% *v/v*. Cells were pelleted by centrifugation for 5 min. Then, 100 µL of supernatant was transferred to a black 96-well plate. EV uptake was quantified by fluorescence using a Tecan plate reader (excitation λ 565 nm, emission λ 615 nm) (Tecan Trading AG, Mannedorf, Switzerland). 

To determine EV uptake in differentiated cells, Caco-2 cells were seeded on Transwell^®^ inserts at 10^5^ cells/well and cultured for 21 days. Caco-2 cell growth, polarisation and cell monolayer integrity were monitored by measuring the transepithelial electrical resistance (TEER). After 21 days, culture medium was removed from inserts and replaced with 0.5 mL of HBSS to equilibrate the cells (45-min incubation). Thereafter, EV uptake was measured by applying EV samples to the apical side of polarised monolayers for 4 h. The applied samples were then removed, cells washed with PBS and subsequently detached from inserts and permeabilised with trypsin and 1% *w/v* Triton X-100. Uptake was determined by fluorescence, which was measured as above. 

### 2.6. Influence of EV Encapsulation on Antiproliferative Effects of CUR

The MTS assay was performed to test the antiproliferative effect of CUR following encapsulation into EVs. Caco-2 cells were seeded on 96-well plates at 10^4^ cells/well and incubated at 37 °C/5% CO_2_ in culture medium for 24 h before the assay. Culture medium was aspirated and replaced with the samples suspended in exosome-depleted DMEM. Exosome-depleted DMEM was used as a negative control and Triton X-100 (1% v/v in exosome-depleted DMEM) as a positive control. Cells were incubated with samples and controls at 37 °C/5% CO_2_ for 72 h. After the incubation period, sample solutions were aspirated and cells washed with PBS. 100 µL of culture medium (DMEM) was added to each well, followed by 20 μL of MTS Reagent. Cells were incubated with the MTS reagent for 3 h at 37 °C. After incubation, the absorbance was measured at 492 nm. The relative cell metabolic activity (%) was calculated using Equation (3):

(3) $$Relative\;metabolic\;activity=\frac{S-T}{D-T\;}\times 100$$

where S is the absorbance with the tested samples, T is the absorbance with Triton X-100, and D is the absorbance with exosome-depleted DMEM

### 2.7. Intestinal Permeability 

Caco-2 cells were seeded and cultured on inserts for 21 days as above. Permeability assays were commenced by replacing the culture medium with 0.5 mL of HBSS and 45-min incubation to equilibrate the cells. Thereafter, EVs were applied to the apical side of cell monolayers for 4 h, followed by periodic sampling from the basolateral side. EV permeability was quantified by fluorescence, measured as above. 

### 2.8. Effect of EV-Incorporation on Intestinal Permeability of CUR

CUR was incorporated into EVs as described above. Permeability study on polarised/differentiated Caco-2 monolayers was conducted as above, with EV-incorporated CUR and free CUR applied to the apical side of cell monolayers at equivalent concentrations. Specifically, CUR concentrations amounted to 15 µg/mL for milk EVs and 8 µg/mL for Caco-2 EVs. Permeability study was undertaken for 4 h with periodic sampling of the basolateral solution and quantitation of CUR. 

### 2.9. Statistical Analysis

Unpaired, unequal variance t test (or Welch t test) was performed for comparisons of two group means, while one way analysis of variance (ANOVA) was utilized for comparison of three or more group means. *P* value of <0.05 was considered statistically significant. ***, ** and * indicate *p* < 0.001, *p* < 0.01 and *p* < 0.05, respectively. Statistical analysis was conducted using GraphPad Prism^®^ Software (version 6, San Diego, CA, USA). 

## 3. Results

### 3.1. Characterisation of EVs 

EVs isolated from cow milk and from Caco-2 cells were initially characterised for hydrodynamic size, polydispersity index and zeta potential. This analysis was conducted for unmodified EVs, fluorescently-labelled EVs, and CUR-incorporated EVs. Data are reported in Supplementary Materials, Table S1. Unmodified milk EVs showed an average diameter of 217 nm, PdI around 0.34 and zeta potential of −8.1 mV. Fluorescently-labelled milk EVs were found to display a larger hydrodynamic radius of 278 nm, and zeta potential of −7.1 mV. CUR-incorporated, fluorescently-labelled milk EVs were found to have hydrodynamic radius of around 390 nm and a zeta potential similar to unloaded, fluorescently-labelled EVs. 

Caco-2-derived unmodified and fluorescently-labelled EVs, with or without CUR, displayed similar hydrodynamic radii and zeta potential values to milk-derived counterparts, specifically 200, 275 and 351 nm, respectively. Zeta potential values for unmodified cell-derived EVs were similar to milk counterparts and somewhat lower for fluorescently-labelled and CUR-incorporated (and labelled) Caco-2 cell-derived EVs compared to systems originating from cow milk. 

**Figure 1:**
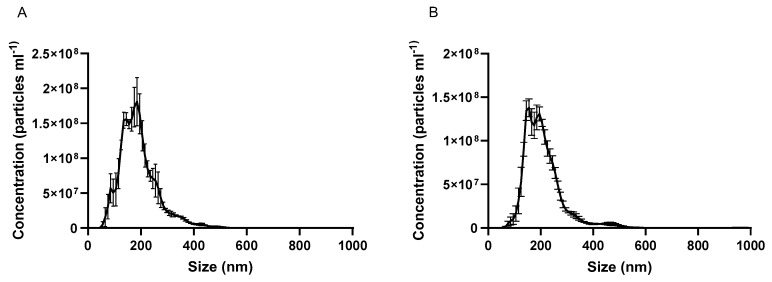
Nanoparticle tracking analysis (NTA) graphs of (**A**) cow milk extracellular vesicles and (**B**) Caco-2 cell-derived extracellular vesicles. Data are shown as mean +/− SD (*n* = 3).

NTA was used as an alternative approach to ascertain the size of unmodified EVs and to establish the yield (nanoparticle numbers/volume). Data are shown in Figure 1A,B for cow milk and cell-derived EVs, respectively. Table 1 shows that NTA data is in agreement with DLS (Supplementary Table S1), with milk-derived EVs found to be 186 nm in size and cell-derived counterparts 204 nm. 

Table 1 additionally depicts the yield and protein content of cow milk and Caco-2 cell-derived EVs. 

### 3.2. CUR Loading and Entrapment Efficiency

Encapsulation efficiency calculated from the elution peaks for milk derived EVs and Caco-2 derived EVs (Supplementary Figure S1) were 36% and 12% (9 µg of CUR per 100 µg of milk EV proteins and 3 µg of curcumin per 100 µg of Caco-2 EV proteins), respectively, while drug loading values were 9% and 3%, respectively. 

### 3.3. Cell Uptake of Evs in Undifferentiated and Differentiated Caco-2 Cells 

**Figure 2:**
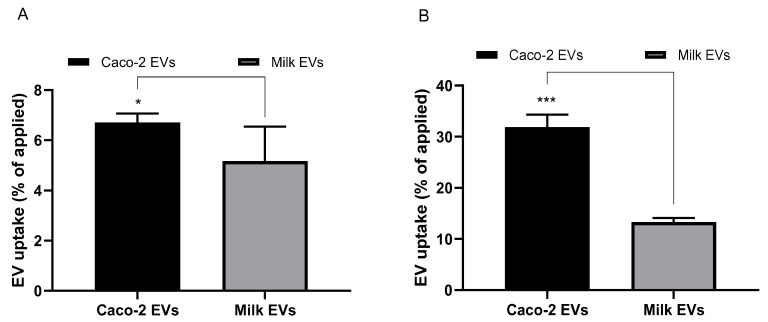
Cell uptake of extracellular vesicles (EVs) (expressed as EV protein amount) in undifferentiated and differentiated Caco-2 cells. (**A**) Cell uptake (percentage of applied dose) of milk-derived and Caco-2 derived EVs in undifferentiated Caco-2 cells cultured on 48-well plates. Cells were incubated with EVs for fourh. Data shown as the mean +/− SD (*n* = 3). * denotes *p* < 0.05. (**B**) Cell uptake of milk-derived and Caco-2-derived EVs in Caco-2 differentiated/polarised cells. Cells were cultured for 21 days on permeable inserts. Cells were incubated with EVs for fourh. Data shown as the mean +/− SD (*n* = 3). *** denotes *p* < 0.001.

The cellular uptake of milk- and cell-derived EVs in undifferentiated and differentiated Caco-2 cells following 4-h incubation is shown in Figure 2 (A and B, respectively). Figure 2A shows that 5% of the applied amount of milk EVs were taken up by cells (48-well plate format). Uptake of cell EVs amounted to 6.7% of the applied dose per well. 

Regarding cell uptake into differentiated Caco-2 cells (polarised monolayers), Figure 2B shows that the uptake of both milk and Caco-2 EVs was markedly higher in differentiated cells compared to non-differentiated cells (both cultured to confluence on substrates of equivalent surface area of 1.1 cm^2^). Specifically, cell uptake of EVs was around two-fold higher (13.31%) for milk EVs and almost five-fold higher (31.83%) for cell-derived systems. Cell EVs seem to be associated with a notably higher level of cell uptake than milk-derived systems. 

### 3.4. Antiproliferative Effects of EV-incorporated CUR

**Figure 3:**
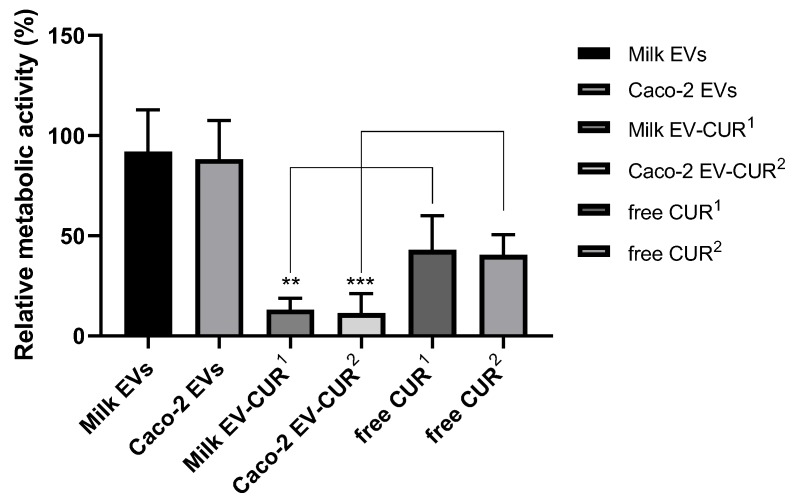
Antiproliferative activity of extracellular vesicle-incorporated curcumin (EV-CUR) and free curcumin (CUR) in Caco-2 cells. Triton X-100 (0.1% *v/v*) and exosome-depleted culture medium were used as controls relative to which metabolic activity of samples was calculated. Cells were treated with milk EV-CUR^1^ (4.5 µg/mL), free CUR^1^ (4.5 µg/mL), Caco-2 EV-CUR^2^ (1.5 µg/mL), free CUR^2^ (1.5 µg/mL). Data shown as the mean +/− SD (*n* = 6). ** and *** denotes *p* < 0.005 and *p* < 0.001, respectively.

Figure 3 shows the impact of EV encapsulation on antiproliferative effects of CUR, as determined by the MTS assay. EV-encapsulated CUR demonstrated a notably greater inhibition of cell proliferation compared to the same applied concentration of free CUR. Specifically, the antiproliferative activity of CUR increased by 34% when incorporated in milk EVs and by 26% following incorporation in cell EVs. Milk and cell EVs alone exhibited no significant inhibition of Caco-2 cell metabolic activity (11% and 14% decrease, respectively). 

### 3.5. Intestinal Translocation of EVs

**Figure 4:**
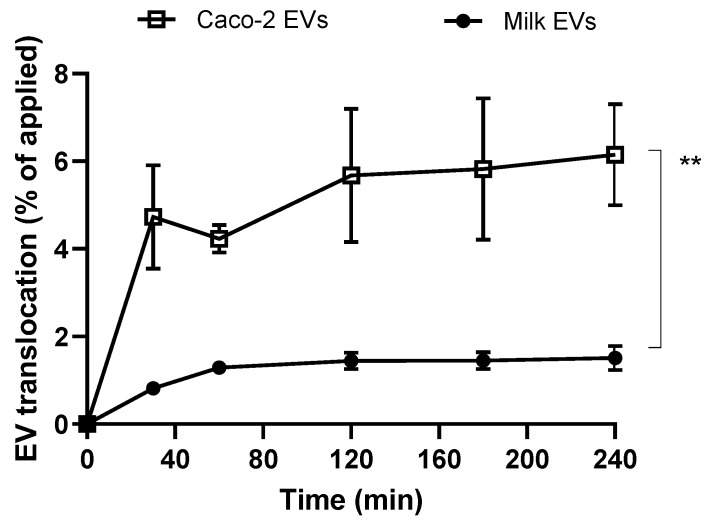
Translocation of milk-derived extracellular vesicles (EVs) and cell-derived EVs in differentiated/polarised Caco-2 cells. Data shown as the mean +/− SD (*n* = 3). ** denotes *p* < 0.01.

Following investigation of cell uptake of milk- and cell-derived EVs, we were interested in comparing their ability and capacity to traverse intestinal epithelial Caco-2 monolayers (intestinal barrier model). Figure 4 depicts that cell-derived EVs demonstrated a markedly higher capacity to translocate across Caco-2 monolayers compared to milk-derived EVs. While there was an overall trend of gradual accumulation of EVs in the basolateral compartment, the rate of EV translocation was highest in the first 30 min. By the last sampling point (4 h), cell EV translocation amounted to 6.15% compared to 1.51% for milk-derived counterparts.

### 3.6. Intestinal Translocation of EV-Incorporated CUR 

Following on from demonstration of ability of milk and cell EVs to translocate across intestinal Caco-2 monolayers, we tested whether intestinal permeability of CUR might be improved by incorporation into these biological vesicles. Table 2 reveals that apical-to-basolateral permeability of CUR (tested over 4 h) was significantly higher following EV-incorporation, as compared to free CUR, for both cell and milk-derived EVs. However, CUR permeability enhancement following incorporation into cell derived EVs was notable more pronounced compared to milk EV-CUR, with enhancement ratio of 5.1 versus 2.9. 

## 4. Discussion

Ingestion is by far the most preferred route of drug administration, but many drugs require parenteral administration (injections) owing to poor oral bioavailability. Low oral bioavailability can result from a number of issues, with key factors involving low water solubility of the drug, instability in the gastrointestinal tract environment, low absorption across the intestinal epithelium, or the ‘first-pass’ metabolism. Particle-based carriers have the potential to overcome some of these issues and improve bioavailability following oral drug administration. However, particles require careful engineering for oral drug delivery as normally most particulates show limited ability to translocate across the gastrointestinal mucosa. 

The ability of EVs, particularly exosomes, to act as the body’s natural delivery systems, effectively transferring cargo from one cell to another, makes them promising natural systems for oral drug delivery. However, it is acknowledged that the current challenge in producing and/or harvesting certain EVs on a commercial scale remains a concern [20,21]. 

Studies have shown that EVs (exosomes) from cow milk are capable of withstanding the hostile gastric conditions, maintaining, and transferring their contents from the gastrointestinal tract into the blood [15,22,23]. A human study demonstrated that functional miRNAs from cow milk are absorbed systemically [22] and studies in mice showed that exosomes from cow milk are capable of cargo delivery across the gastrointestinal epithelium [15,16,23,24], with therapeutic applications including targeted delivery to cancer. 

This work for the first time probes the oral drug delivery enhancing potential of intestinal epithelial cell-derived EVs, whilst providing a comparison of this potential with cow milk-produced EVs. EVs were successfully isolated, characterised, labelled and loaded with a poorly absorbable compound serving as a model drug (CUR) (Figure 1 and Supplementary Figure S1, Table 1 and Supplementary Table S1). We then performed cell uptake and transport analyses in undifferentiated and differentiated intestinal Caco-2 cells. 

Cell uptake data reveal that while both milk- and intestinal epithelial cell-derived EVs were taken up by undifferentiated and differentiated (polarised monolayer) intestinal Caco-2 cells (Figure 2A,B, respectively), Caco-2-derived EVs demonstrated a higher cell uptake. Interestingly, the uptake of both cow milk and intestinal epithelial cell EVs was notably higher in differentiated Caco-2 cells compared to undifferentiated cells, specifically 2-fold higher for milk EVs and 5-fold higher for cell-derived counterparts. Both milk and cell-derived EVs displayed notably higher levels of cell uptake than, for example, 100 nm unmodified polystyrene nanoparticles reported in our previous study [11]. Specifically, this difference amounts to 5% and 6.7% cell uptake for milk and cell EVs, respectively, versus 1.57% for polystyrene nanoparticles in undifferentiated cells, whereas in polarised monolayers cell uptake of milk and cell EVs was observed to be 13.31% and 31.83%, respectively, versus 4.1% with polystyrene nanoparticles.

A recent study has demonstrated that the neonatal Fc receptor (FcRn)-a receptor responsible for trafficking of immunoglobulin G (IgG) across epithelia, including the intestinal epithelium-may play a critical role in the intestinal absorption of cow milk exosomes [13]. Specifically, in a competition experiment in mice were co-administered exosomes with varying amounts of free bovine IgG, which reduced the uptake of exosomes. If the intestinal absorption of exosomes is indeed mediated via FcRn, this may explain the significantly higher uptake of EVs in differentiated Caco-2 cells. This is because the expression level of FcRn is differentiation-dependent, with the level of FcRn expression in post-confluent Caco-2 cells found to be approximately three-fold higher compared to that in pre-confluent cells [25]. 

To demonstrate that the cell uptake-enhancing effect of EVs can be utilised to facilitate the intracellular delivery of drugs, we used CUR as a model drug with poor cellular uptake [26]. Studies determining the anti-proliferative effect showed that EV-incorporated CUR was associated with a significantly higher suppression of cell proliferation compared to free CUR, with its antiproliferative activity increasing by 34% following encapsulation in milk EVs and by 26% when presented to the cells incorporated in intestinal epithelial cell-derived EVs (Figure 3). This is in agreement with a previous study evaluating the antiproliferative activity of EV-encapsulated CUR against several human cancer cell lines (lung, cervical, and breast cancer cells), which demonstrated a significantly increased antiproliferative efficacy of EV-enclosed CUR (compared to free CUR) in all the examined cells [27]. 

There have been some attempts to compare the mechanisms of intestinal epithelial trafficking of EVs. A prior study utilised inhibitors of endocytosis (cytochalasin D) and intracellular vesicle trafficking (brefeldin A) to test the intestinal uptake of microRNAs encapsulated in bovine milk EVs (exosomes) in Caco-2 cells. The attenuation of uptake under these conditions suggested that as an active and saturable process, exosome uptake is most likely mediated by endocytosis [28]. In another study, milk exosome uptake was confirmed to be an energy-dependent process [27]. In addition, pharmacological inhibitors of caveolae-mediated uptake resulted in around 50% decrease in uptake of exosomes in H1299 cells and microtubule inhibitors attenuated the uptake to a lower extent. 

Studies assessing the transepithelial transport of EVs across polarised (differentiated) Caco-2 monolayers show that intestinal epithelial cell-derived systems demonstrated a significantly more pronounced ability to translocate across the intestinal model (Figure 4). The data therefore suggest that intestinal epithelial cell-derived EVs would potentially show more prominent bioavailability-enhancing effects compared to cow milk EVs. 

A previous study demonstrated that encapsulation of CUR in milk EVs protected this compound from gastrointestinal digestion (tested in vitro) and facilitated its permeability across Caco-2 monolayers compared to free CUR [29]. Here we confirm these findings (Table 2). However, in line with observations that intestinal epithelial cell-derived EVs permeated Caco-2 monolayers to a greater extent compared to cow milk counterparts, Table 2 also confirms that CUR permeability across the Caco-2 intestinal model is enhanced significantly more following incorporation in intestinal epithelial cell-derived EVs relative to cow milk EV. 

Overall, this study confirms the ability of cow milk EVs to traverse the intestinal epithelial barrier, but shows that EVs derived from intestinal epithelial cells themselves have a notably superior capacity to overcome this barrier. Cow milk derived EVs are more attractive potential delivery systems due to lower production/scalability cost. Additionally, it has been demonstrated that cargo (dietary microRNAs) encapsulated in milk exosomes is protected against degradation by low pH, RNases and treatment that mimics digestion in the gastrointestinal tract [23,30,31], making cow milk exosomes potentially attractive systems for oral drug delivery. However, the greater intestinal epithelium barrier crossing capacity of EVs from human intestinal epithelial cells may be more useful and produce a more pronounced improvement of oral drug bioavailability. At the same time, the stability of intestinal epithelial cell-derived EVs in the gastrointestinal biofluid is not known and was not assessed in this study. Future work should further explore their potential utility for facilitating oral drug delivery, particularly of macromolecules, with studies on the optimisation of drug loading (which is particularly challenging with large molecules), assessment of stability of EVs in the gastrointestinal tract, and determination of epithelial trafficking mechanisms. Finally, the delivery potential and safety of these systems should be confirmed in vivo.

## Figures and Tables

**Table 1:** Size (nm), yield (particles/mL) and protein concentration (µg/mL) of Caco-2 extracellular vesicles and milk extracellular vesicles, as measured using nanoparticle tracking analysis instrument (data expressed as mean +/−SD; *n* = 3). Protein concentration measured using the Bicinchoninic acid (BCA) assay.

EV Type	Size (nm)	Yield (Particle/mL)	Protein (µg/mL)
Caco-2 cells	203.9 ± 4.1	1.82 × 10^9^ ± 1.06 × 10^8^	468.1 ± 15.5
Cow milk	185.8 ± 6.2	2.42 × 10^9^ ± 6.86 × 10^7^	3290 ± 259.3

**Table 2:** Permeability of curcumin (CUR) and CUR incorporated into milk-derived and Caco-2 cell-derived extracellular vesicles (‘Milk EV-CUR’ and ‘Caco-2 EV-CUR’, respectively). Permeability was tested over fourh, with regular sampling of the basolateral solution and CUR quantitation by absorbance. Permeability expressed as apparent permeability coefficient (P_app_). Data shown as the mean +/− SD (*n* = 3). * denotes *p* < 0.05 (EV-CUR vs. ‘Free CUR’).

Sample	CUR Permeability, P_app_ (cm/s) (±SD)	Enhancement Ratio
Free CUR	2.69 × 10^−6^ (±3.84 × 10^−7^)	-
Milk EV-CUR	7.70 × 10^−6^ (±3.93 × 10^−6^) *	2.9
Cell EV-CUR	1.38 × 10^−5^ (±1.12 × 10^−6^) *	5.1

## Supplementary Materials

The following are available online at https://www.mdpi.com/1999-4923/12/3/226/s1, Figure S1: Size exclusion chromatography profiles, Table S1: Hydrodynamic size, polydispersity index and zeta potential of EVs.
