# Supplementary material for: Cow Milk and Intestinal Epithelial Cell-Derived Extracellular Vesicles as Systems for Enhancing Oral Drug Delivery
Source: Pharmaceutics. 2020 Mar 4;12(3):226. doi: 10.3390/pharmaceutics12030226 (PMC7150822; doi:10.3390/pharmaceutics12030226)
Supplement: Supplementary file 1 [file pharmaceutics-12-00226-s001.pdf]

Supplementary Information - Cow Milk and Intestinal Epithelial Cell-derived Extracellular Vesicles as Systems for Enhancing Oral Drug Bioavailability

Characterisation of EVs

Hydrodynamic size, polydispersity index and zeta potential of EVs is shown in Table S1.

EV type	Milk unmodified	Milk labelled	Milk labelled CUR-incorporated	Cell unmodified	Cell labelled	Cell labelled CUR-incorporated
**Size (nm)**	217.3 ± 23.8	277.5 ± 5.4	387.9 ± 70.1	200.2 ± 23.7	274.5 ± 14.1	351.2 ± 14.1
**Zeta potential**	-8.1 ± 1.4	-7.1 ± 1.7	-7.2 ± 3.8	-8.4 ± 1.5	-5.6 ± 1.4	-5.5 ± 1.4
**PdI**	0.34 ± 0.04	0.34 ± 0.04	0.38 ± 0.03	0.37 ± 0.05	0.34 ± 0.03	0.36 ± 0.04

***Table S1**. Hydrodynamic size, zeta potential and polydispersity index of cow milk- and Caco-2 cell-derived extracellular vesicles, as determined by dynamic light scattering.*

CUR Loading and Entrapment Efficiency

The incorporation of curcumin into EVs was performed via overnight incubation (with stirring) of EVs with CUR at EV protein to CUR ratio of 1:4. Unencapsulated CUR was removed by size exclusion chromatography, using a PD-10 column prepacked with Sephadex G-25. Elution profiles are shown in Supporting Information, Figure S1. 1 ml fractions were collected from the elution of controls (CUR alone and EVs alone, Figure S1A) and loaded EVs (Figure S1B) and for each fraction CUR concentration was determined from absorbance at 430 nm against a calibration curve. Free and non-incorporated CUR eluted in fractions 9, 10 and 11 in both controls and loaded EVs, whereas, due to the large size of the vesicles, incorporated CUR eluted in fractions 2 and 3 in both milk and Caco-2 EVs, allowing good separation from excess free CUR.

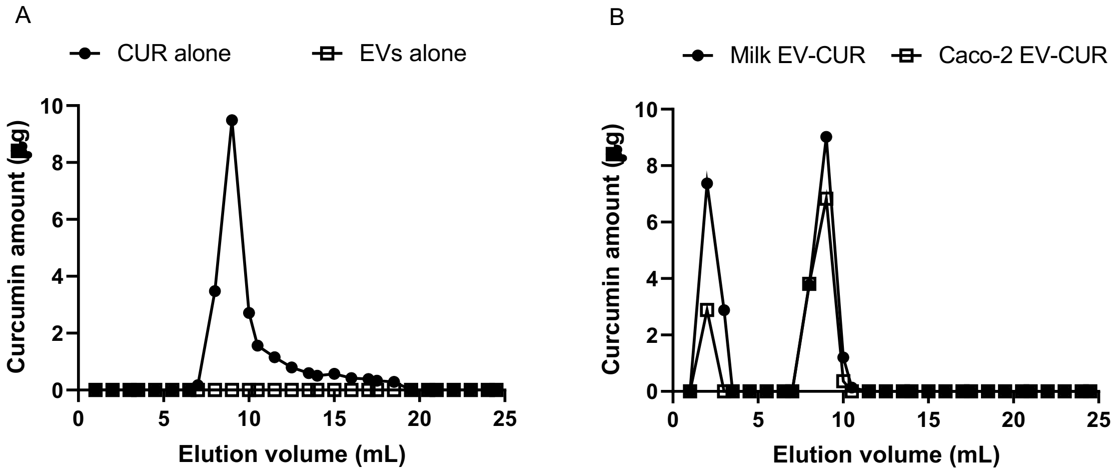

***Figure S1**. Size exclusion chromatography profiles. A) elution profile of controls: curcumin alone ('CUR alone') and extracellular vesicles alone ('EVs alone'). B) Elution profiles of milk EV-incorporated CUR ('milk EV-CUR') and Caco-2-derived EV-incorporated CUR ('Caco-2 EV-CUR').*
